# Behavioral Model of Middle-Aged and Seniors for Bicycle Tourism

**DOI:** 10.3389/fpsyg.2020.00407

**Published:** 2020-04-09

**Authors:** Shu-Wang Lin, Shih-Yun Hsu, Juei-Ling Ho, Mei-Ying Lai

**Affiliations:** ^1^Office of Physical Education, Chienkuo Technology University, Changhua, Taiwan; ^2^Department of Business Management, National Taichung University of Science and Technology, Taichung, Taiwan; ^3^Department of Hotel Management, Tainan University of Technology, Tainan, Taiwan; ^4^Department of Leisure Regimen Management, Tainan University of Technology, Tainan, Taiwan

**Keywords:** sport habit, attitude, subjective norm, perceived behavioral control, behavioral intention, theory of planned behavior, national scenic area

## Abstract

This research centers on the behavioral tendency of the middle-aged and seniors in bicycle tourism at environmentally protected scenic areas and its relevant influence factors. The theory of planned behavior (TPB) is adopted as the basis of this study. The middle-aged and seniors are the subjects of this research. A questionnaire survey is conducted at environmentally protected national scenic areas in Taiwan. A total of 230 samples are drawn with a random sampling method, and 210 are valid. The findings indicate two things. First, when applying the TPB to different fields of the study, the level of predictability may vary. Another finding is that subjective norm shows a higher level of susceptibility to sport habit and predictability to behavioral intention than the other two constructs. With an empirical analysis, the study is able to provide middle-aged and senior participants and sport administration authorities with relevant suggestions for reference at the end of this paper.

## Introduction

According to the 2018–2065 Population Projections for Taiwan conducted by the National Development Council (2018), the number of the aged population (65 and older) had exceeded the young age population (0–14) in 2017; by 2065, the aging index will climb to 450.1 (i.e., the aged population will be 4.5 times the young population) exhibiting the aging trend of Taiwan’s population structure. Currently, the pursuit of better life quality is a tenet among the public. Seeking mental and physical fitness along with life satisfaction has become the norm. Huang (2006) indicates that leisure sports can increase personal life satisfaction and subjective well-being while bringing individuals and society multiple benefits, such as maintaining physical fitness, dispelling fatigue, relieving stress, and achieving self-affirmation. In terms of social benefits, leisure sport can promote harmonic interpersonal relationships and social networking. The right to and opportunities for leisure sports are for people of all ages. In the Taiwanese society that promotes “Sports for All,” not only the youth are enthusiastic regarding various types of exercises, but also the middle-aged (45–64) and seniors (65 and older) are encouraged to participate in leisure sports. It echoes what Huang (2006) indicates above. This helps them adapt to their aging status, realize an ideal life in their old age, and, in turn, reduce social problems and costs.

In recent years, cycling sport has gained popularity among the public. Some cities in Taiwan have already established public bicycle rental systems. The broadly established bicycle paths provide the public with more access to cycling. The “Integrated Bicycle Path Network Construction Plan” in 2012, promoted by the Sports Administration, Ministry of Education (Sports Administration Ministry of Education, 2012), points out that in response to the growing popularity of leisure sports, the National Development Council has followed the guidance of the “Economic Revitalization Project with Investment Expansion (NT500 Billions in 4 Years) in Public Works” to construct the around-the-island bicycle paths by establishing interconnected regional networks. Through the subsidies of the “Integrated Bicycle Path Network Construction Plan” to the local governments from 2012 to 2016, high-quality bicycle paths fitted with local terrains are constructed in order to meet the demands in leisure activities and sports (Maier and Weber, 1993). The plan attests to the government’s proactive promotion in the cycling sport. On the other hand, when cycling is combined with tourism, a new phenomenon of sport tourism is henceforth created: bicycle tourism (Beech et al., 2005). In 2017, around 2.5 million people are involved in bicycle tourism in Taiwan (National Development Council, 2018). Whether bicycle tourism is of participation, sightseeing, or nostalgia type, it has already attracted specific groups of people. With the change of physical functions among the middle-aged and seniors, appropriate exercise can help offer certain positive effects concerning their mental and physical status.

Tsai and Chou (2008) argue that the increased muscle strength could allow seniors to partake in more aerobic exercises, such as hiking and cycling. The fact validates the appropriateness of cycling sport for the middle-aged and seniors. Furthermore, bicycle tour itineraries offered by many tourist attraction authorities or travel agencies in recent years have helped to enhance their cognition toward this tourism phenomenon (Fotiadis et al., 2016a). For example, in 2014, the Sun Moon Lake National Scenic Area Administration (2014) introduced a 1.5-km bicycle/hiking path to connect the scenic spots of Wenwu Temple, Songbolun, and Dazhuhu in order to promote relevant bicycle tour itineraries and friendly facilities for seniors. Through hiking or cycling, tourists are immersed in nature and health atmosphere of the ancient salt-transporting trail at Songbolun. During the journey, tourists are able to take in the mountain and waterfront scenery along with the eco-diversity that the surrounding environment offers. It is noted that the bicycle path network development conducted by the government also allows more access for the middle-aged and seniors to engage in bicycle tourism.

Past studies on the sport participation of the middle-aged and seniors mainly focused on Tai chi (an internal Chinese martial art), Yuanji dance, Waidangong, and other leisure exercises (Yang, 2001; Chung et al., 2005; Huang, 2005; Hung, 2005; Wu, 2010; Chang W.H. et al., 2014; Vassiliadis and Fotiadis, 2014), while a minor portion of them explore the issues of water sports (Chien et al., 2012; Huang et al., 2012). In addition, Lin and Wu (2010) investigated the effect of walking exercise on cardiovascular risk factors of the middle-aged and seniors in communities. However, researches on the participation in cycling sport among the middle-aged and seniors are still relatively rare.

In light of the growing popularity of bicycle tourism and the comments of some scholars that previous researches largely focus on “who” participate in sport tourism, rather than “why” participate (Gibson, 2005), this research centers on the behavioral tendency of the middle-aged and seniors in bicycle tourism at environmentally protected scenic areas and its relevant influence factors. The theory of planned behavior (TPB) is adopted as the basis of this study. With an empirical analysis, the study is able to provide middle-aged and senior participants and sport administration authorities with relevant suggestions for reference at the end of this paper.

The paper below is organized in the following way. *Literature Review* introduces the sport tourism and the TPB used in this paper. *Research Methodology* provides research framework and hypothesis, measurement, sampling site, sampling method, and respondents’ profile of this research. *Results* includes the examination of offending estimate, confirmatory factor analysis, reliability and validity, and hypotheses testing. *Conclusions, Contribution, and Suggestions* presents the conclusions, theoretical contributions, empirical suggestions, and suggestions for future research of this study.

## Literature Review

### Sport Tourism

The United Nations World Tourism Organization (UNWTO), 2008 defines tourism as follows: “Tourism is a social, cultural and economic phenomenon which entails the movement of people to countries or places outside their usual environment for personal or business/professional purposes. These people are called visitors (which may be either tourists or excursionists; residents or non-residents) and tourism has to do with their activities, some of which imply tourism expenditure.” Sport tourism is the participation of people in sport-related activities for a certain period of time in an environment outside their usual place of residence (Ritchie and Adair, 2002; Pigeassou, 2004; Bartolomé et al., 2009; Weed, 2009; Fotiadis et al., 2016b, c). There are several categorizations of sport tourism. Gammon and Robinson (Gammon and Robinson, 2003) recommend that sports tourism can be classified as hard sports tourism or soft sports tourism, while Gibson (2006) recommends that there are three categories of sports tourism: sports event tourism, celebrity and nostalgia sport tourism, and active sport tourism. Kazimierczak and Malchrowicz-Mosko have introduced specific and developmental trends of sport tourism and presented deeper analysis about the essence of sport tourism.

The hard definition of sport tourism refers to the mass of people participating at competitive sport events. Normally in these kinds of sport events, enthusiasm attracts visitors to the events. FIFA World Cup, Olympic Games, F1 Grand Prix, and local events such as Half-Marathon and NASCAR Sprint Cup Series could be expressed as hard sports tourism (Grabowski, 1999; Gammon and Robinson, 2003; Pigeassou, 2004; Tyrrell et al., 2004; Weed, 2009). The soft definition of sport tourism is when the visitors travel to take part in recreational sporting, or sign up for leisure interests. Hiking, running, skiing, bicycling, and canoeing can be expressed as soft sports tourism (Gammon and Robinson, 2003; Pigeassou, 2004; Weed, 2009). In this study, bicycle tourism is a kind of soft sport tourism.

According to Gibson (1998), activities that sport tourists partake can be categorized into three types depending on an individual’s main purpose. Tourists can either be a participant of sport or a spectator, or a visitor of sport-related sites. This study, obviously, focuses on the first type of sport tourists, namely, the active sport participant (Gibson, 1998), with a slight distinction. Most studies on the motivation of an active sport participant mainly focus on younger people, for whom competing is a major motive. As proven by some studies, young athletes show a higher level of aggressive tendency (e.g., Keskin, 2018) and are prone to risk taking (Blazo and Smith, 2018). The main subject of this study aims at middle-aged to senior respondents. Their motivation for participating in sport activity is likely to be different than their younger counterparts (Gayman et al., 2017), as well as their concerns (Johnson and Rose, 2015).

### Theory of Planned Behavior

In investigating people’s behavior, the major part of the discussion on behavioral intention focuses on the “will” or “will not” of performing a specific behavior (Ajzen and Fishbein, 2005). The TPB bases the prediction of behavioral intention upon personal attitude, subjective norm, and perceived behavior control; behavioral intention is the best variable for behavior prediction (Ajzen, 1985). The TPB originates from the theory of reasoned action (TRA) proposed by Fishbein and Ajzen (1975); its fundamental assumption is that “human behavior is based on rational reasoning, and an individual can appropriately control his or her behavior with personal will.” However, in reality, not all behaviors are subject to personal will; instead, they are also influenced by the external objective environment or the resource limitation. For example, the rather costly registration fee or not having the time for a bicycle sport event can determine individuals’ participation decisions. Factors of this nature make TRA inapplicable in explaining sport participation. It is in this circumstance that Ajzen (1985) adds the “perceived behavioral control” variable into the TRA model via an integrated review of relevant social psychological literature. In the TPB developed by Fishbein and Ajzen (1975), “attitude” is a personal positive or negative perception or belief about a specific behavior. It is formed through the conceptualization of the judgment about a specific behavior.

“Subjective norm” is the social pressure perceived by an individual about a specific behavior he/she performs. However, “perceived behavioral control” is the level of control about a specific behavior, as perceived by the individual, who can even anticipate the probable impediments and obstacles of performing this behavior according to his/her past experience. Take this research as an example: if the middle-aged and seniors believe that cycling sport can enhance their health, and they deem this to be a very important positive consequence, then their attitude toward regular exercise will tend to be positive accordingly. In addition, if their relatives or friends approve of this idea that cycling sport is healthful, and the middle-aged and seniors are also willing to take their advice, the “subjective norm” in this case will be strong. Finally, if, most of the time, they cannot find companions to do cycling, and companions are a significant factor for their participation in cycling, they will regard “lack of a companion” as an obstacle; this in turn will consequently influence their behavioral intention about cycling. However, the TPB also has its own limits. Hsu (2006) points out that the TPB could present good predictions while explaining the relationship between intention and behavior; however, research result inconsistency still occurs in the case of different research subjects, age groups, and behaviors probed in different research. This is where extra attention is required during the research process. Furthermore, the influence of a regular exercise habit on behavioral intention is corroborated by relevant research. In their study, Kaushal et al. (2017) indicate that a regular exercise habit is the most important factor determining whether or not an individual will continue to exercise regularly. A study on military personnel’s sport attitude, values, and intention to participate in exercises by Chen (2008) also verifies that the “regular exercise factor” scored the highest in the sport behavioral intention. Accordingly, sport habits have a significant influence on the behavioral intention; their relation will be discussed in this paper.

From the related literature, it can be learned that the TPB has been applied to relevant research on exercise by seniors. Dean et al. (2007) examine the physical fitness training of the seniors through the TPB. Conn et al. (2003) employ the TPB to investigate the sport behavior of female seniors. Gretebeck et al. (2007) adopt the TPB to discuss the physical activities and body functions of the seniors. The TPB has also been long applied to the research on sport participation (Hamilton and White, 2008) and tourism activities (Quintal et al., 2010), thereby indicating its applicability to research on sport tourism issues. Ajzen and Driver (1992) divide the behavior to be predicted into two categories: daily activity (such as biking) and less-participated activity (like mountain climbing). According to their research results, perceived behavioral control could effectively predict the behavioral intention concerning daily activities, but failed to predict the behavior itself. Contrarily, for the less-participated behavior, the perceived behavioral control could present good predictions for both behavioral intention and the behavior itself. This discovery elicited from the literature review constitutes the motivation for this research. Therefore, this study will extend the TPB model by adding a new factor of sport habit and taking the middle-aged and seniors as the research subjects to investigate their behavioral intention of participation in bicycle tourism.

### Bicycle Tourism at the National Scenic Areas in Taiwan

**MAP 1:**
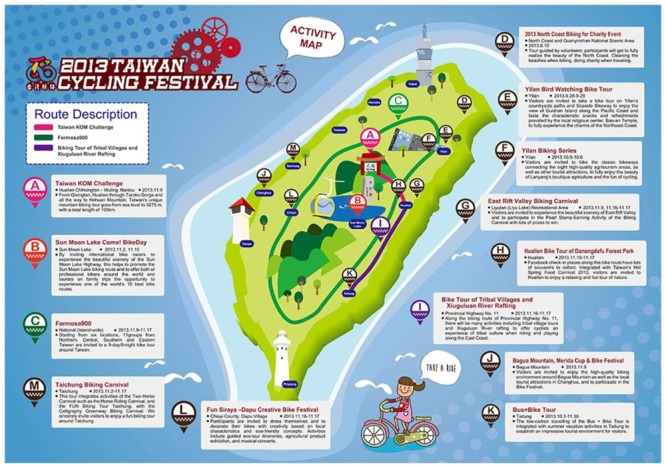
Biking map for Taiwan Cycling Festival. *Source*: Taiwan Tourism Bureau, Republic of China (Taiwan) (2019).

**MAP 2:**
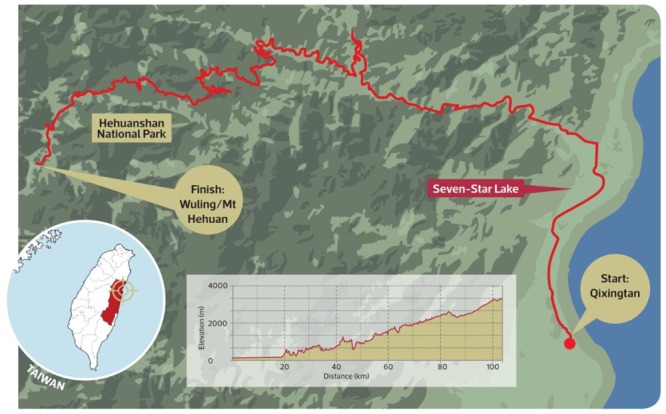
Biking map for Taiwan KOM (King of Mountain) Challenge. Sources: Taiwan Tourism Bureau, Republic of China (Taiwan) (2019).

Bicycle tourism is prosperous in Taiwan. According to the National Development Council (2018), around 2.5 million people are involved in bicycle tourism in 2017. Thus, bicycle tourism is one of important sport tourisms in Taiwan. The Taiwan Tourism Bureau has promoted bicycle tourism intensively and developed various cycling routes. For examples, the Taiwan Tourism Bureau has promoted Creative Cycling Routes, Top 10 Cycling Routes, Taiwan Cycling Festival (please see Map 1), and Taiwan KOM (King of Mountain) Challenge (please see Map 2). Inevitably, the Taiwan Tourism Bureau has also developed cycling routes at its national scenic areas (Tourism Bureau, Republic of China (Taiwan), 2019).

The national scenic areas in Taiwan are managed by the Taiwan Tourism Bureau. The National Scenic Area is established according to Article 11 of the “Statute for the Development of Tourism,” and Articles 18 and 19 also point out the areas with beautiful landscape, ecology, culture, and humanistic tourism value of nature, which should be planned to be established as national scenic areas, prohibited from destruction of relevant ecological tourism resources, and maintained for the sustainable development of natural resources. Currently, there are 13 national scenic areas in Taiwan (Bureau).

Bicycle tourism is one of the tourism activities preferred by the middle-aged and seniors in Taiwan. These middle-aged and seniors have time and money to be involved in the activities at national scenic areas (Tourism Bureau, Republic of China (Taiwan), 2019). Therefore, bicycle tourism at environmentally protected national scenic areas in Taiwan becomes an important issue of this study.

## Research Methodology

### Research Framework and Hypothesis

According to the literature review regarding the sport tourism and the TPB, research objectives, and the research framework, the following hypotheses are proposed:

H1: Sport habit has a positive impact on a respondent’s attitude toward biking.

H2: Sport habit has a positive impact on a respondent’s subjective norm toward biking.

H3: Sport habit has a positive impact on a respondent’s perceived behavioral control toward biking.

H4: Attitude has a positive impact on a respondent’s behavioral intention toward biking.

H5: Subjective norm has a positive impact on a respondent’s behavioral intention toward biking.

H6: Perceived behavior control has a positive impact on a respondent’s behavioral intention toward biking.

**FIGURE 1:**
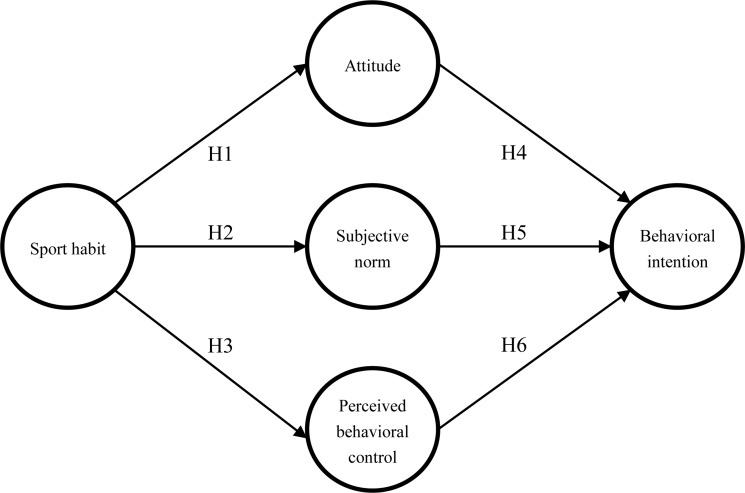
Research framework.

Based on the TPB and the principles of establishing a theoretical framework, and the relation among hypotheses, a research framework was developed, as shown in Figure 1.

### Measurement

The behavioral tendency scale used in this research was adapted from past studies of sport behavior (Huang, 2005; Lin, 2006; Lo, 2007) and the TPB (Ajzen and Fishbein, 1980; Ajzen, 1991; Han and Kim, 2010). The sport habit scale was compiled according to past studies (Zhang, 2002; Guo, 2003; Tsai, 2006). The behavioral tendency scale has 16 questions in total, categorized into four parts: attitude, subject norm, perceived behavioral control, and behavioral intention. The sport habit scale has five questions. All the questions are five-point Likert scale. The respondents answered the questions based on their perception in the following five levels: strongly agree, agree, neutral, disagree, and strongly disagree, with scores of 1, 2, 3, 4, and 5, respectively. Regarding the mean and standard deviation of all questions of the measurement, please see Table 1.

**TABLE 1:** Means and standard deviation of all questions of the measurement.

**Questions**	**All**		**M-age**	**Senior**	**Male**	**Female**
	**Mean**	**SD.**	**Mean**	**Mean**	**Mean**	**Mean**
**Attitude**						
A1. Bicycle tourism is good for me	1.86	0.68	1.80	1.92	1.75	1.97
A2. Bicycle tourism is pleasurable for me	1.96	0.66	1.86	2.07	1.86	2.07
A3. Bicycle tourism is enjoyable for me	2.01	0.73	1.95	2.07	1.89	2.14
A4. Bicycle tourism is fun for me	2.00	0.72	1.92	2.10	1.92	2.09
**Subjective norm**						
B1. My family thinks I should be involved in bicycle tourism	2.47	0.83	2.43	2.51	2.33	2.62
B2. My friend thinks I should be involved in bicycle tourism	2.43	0.82	2.38	2.50	2.25	2.65
B3. My sport companions think I should participate in bicycle tourism	2.40	0.87	2.34	2.46	2.21	2.61
B4. I think I should be involved in bicycle tourism	2.19	0.76	2.15	2.24	1.98	2.43
**Perceived behavioral control**						
C1. I have enough sport resources to participate in bicycle tourism	2.58	0.98	2.38	2.83	2.32	2.89
C2. I have enough energy to participate in cycling tours	2.41	0.91	2.28	2.57	2.16	2.69
C3. I have enough skills to participate in cycling tours	2.55	0.99	2.39	2.74	2.24	2.91
C4. I have enough time to participate in cycling tours	2.62	0.95	2.54	2.72	2.44	2.84
**Behavioral intention**						
D1. In the future I may continue to participate in bicycle tourism	2.91	1.05	2.80	3.04	2.67	3.19
D2. In the future I want to participate in bicycle tourism again	2.64	1.05	2.63	2.65	2.38	2.95
D3. In the future I have plans to participate in bicycle tourism	2.74	1.03	2.64	2.85	2.48	3.03
D4. In the future I plan to participate in different types of bicycle tourism	2.99	1.11	2.82	3.19	2.71	3.31
**Sport habit**						
E1. Participating in bicycle tourism becomes a routine activity in my daily life	2.83	1.1	2.72	2.96	2.58	3.12
E2. Participating in bicycle tourism is a natural thing for me	2.48	0.9	2.37	2.63	2.21	2.80
E3. I will automatically participate in bicycle tourism spontaneously	2.46	0.87	2.32	2.63	2.25	2.70
E4. I often participate in bicycle tourism	2.56	0.93	2.43	2.72	2.35	2.80
E5. I will feel strange if I don’t get involved in bicycle tourism for a long time	2.58	0.96	2.41	2.78	2.34	2.85

*M-age, middle-aged; the pairs of numbers underlined are significantly different.*

### Sampling Method

The middle-aged and seniors at environmentally protected scenic areas in Taiwan are the subjects of this research. A questionnaire survey is conducted at Sun Moon Lake National Scenic Area, East Coast National Scenic Area, and Northeast and Yilan Coast National Scenic Area. The surveys are conducted with face-to-face questionnaire interviews at the visitor centers of the administration offices of the above three national scenic areas. All interviewers are trained for the surveys. The population is the middle-age and seniors who participated in the cycling tours hosted by the Giant Adventure (a travel agency operated by Giant, one of the top 10 bicycle producers in the world). Convenient sampling was used to generate a total of 230 samples. The interviewers will approach every third tourist who will walk into the centers and ask him/her to participate in the survey. A total of 220 questionnaires are returned for a response rate of 95.6%, of which 210 are valid, for an effective response rate of 95.4%.

## Results

### Respondent Profiles

Among the 210 valid samples, 112 are males and 98 are females, which account for 53.3 and 46.7% of the samples, respectively. As for the age distribution, the smallest age group, 75 and older, takes up only 3.8% of the effective samples, while the “45–54” age group and the “55–64” age group are the top two categories, which account for 51.9% and 33.3% of the effective samples, respectively. The remaining “65–74” age group accounts for 11.0% of the effective samples.

With respect to the education level, 40.0% of the effective samples have a “college degree,” the biggest category, followed by 24.8%, the “general and vocational high school” category, while the smallest one is the “elementary school” category, 5.2%. Therefore, most of those middle-aged and senior subjects have a college degree. For the “Annual frequency of participating in sport tourism,” the “less than one time (included)” group is the largest, which accounts for 58.6% of the effective samples, while the “Four times” and “Five times” are the smallest two groups, with both merely 5.7%, indicating that most of the subjects participate in the sport tourism only once a year.

### Examination of Offending Estimate

From Table 2, it is concluded that the error variances of this research are non-negative, ranging from 0.02 to 0.10, and the standardized regression coefficients are distributed from −0.04 to 0.91. This result consists with the offending estimate examination criteria raised by Hair et al. (2016): (1) whether or not the negative error variance exists and (2) whether or not the standardized regression coefficient is higher than or too close to 1.0. The result shows that no offending estimate exists, allowing the overall model fit to be examined.

**TABLE 2:** Test results of offending estimate.

**Questions**	**Standardized**	**Standard**
	**coefficient**	**error**
1. A1 ← Attitude	0.76	0.03
2. A2 ← Attitude	0.88	0.02
3. A3 ← Attitude	0.87	0.03
4. A4 ← Attitude	0.87	0.03
5. B1 ← Subjective norm	0.78	0.03
6. B2 ← Subjective norm	0.89	0.02
7. B3 ← Subjective norm	0.89	0.03
8. B4 ← Subjective norm	0.78	0.03
9. C1 ← Perceived behavioral control	0.82	0.03
10. C2 ← Perceived behavioral control	0.86	0.03
11. C3 ← Perceived behavioral control	0.85	0.03
12. C4 ← Perceived behavioral control	–0.04	0.10
13. D1 ← Behavioral intention	0.91	0.02
14. D2 ← Behavioral intention	0.83	0.04
15. D3 ← Behavioral intention	0.91	0.02
16. D4 ← Behavioral intention	0.90	0.03
17. E1 ← Sport habit	0.88	0.03
18. E2 ← Sport habit	0.87	0.04
19. E3 ← Sport habit	0.90	0.03
20. E4 ← Sport habit	0.88	0.04
21. E5 ← Sport habit	0.85	0.05

### Reliability and Validity

#### Measurement Model Analysis

According to the suggestions of Bagozzi and Yi (1988), the convergent validity of the measurement model is examined using the standardized path coefficient, the average variance extracted (AVE), and the composite reliability in order to conduct the analysis of the measurement model and the associated examination with convergent validity and discriminant validity.

##### Composite reliability and convergent validity

This research examines the convergent validity of the measurement model using the standardized path coefficient, the AVE, and the composite reliability. Generally speaking, the composite reliability should be >0.60, and the AVE should be >0.50 (Fornell and Larcker, 1981).

The standardized parameter estimates used in the confirmatory factor analysis associated with behavioral tendency are listed in Table 3: for the attitude dimension, the factor loading ranges from 0.76 to 0.88; for the subjective norm dimension, it is from 0.78 to 0.89; for the perceived behavioral control dimension, it varies between 0.82 and 0.86; and that for the behavioral intention dimension, it is between 0.83 and 0.91. For the four latent variables, i.e., attitude, subjective norm, perceived behavioral control, and behavioral intention, the composite reliability is 0.91, 0.90, 0.88, and 0.93, respectively, while their AVE is 0.72, 0.70, 0.71, and 0.79, respectively.

**TABLE 3:** Composite reliability and convergent validity.

**Latent variable**	**Factor**	**Composite**	**Average**	
	**variable**	**loading**	**reliability variance**	
				**extracted**
Attitude	A1	0.76	0.91	0.72
	A2	0.88		
	A3	0.87		
	A4	0.87		
Subjective norm	B1	0.78	0.90	0.70
	B2	0.89		
	B3	0.89		
	B4	0.78		
Perceived behavioral control	C1	0.82	0.88	0.71
	C2	0.86		
	C3	0.85		
	C4	–0.04		
Behavioral intention	D1	0.91	0.93	0.79
	D2	0.83		
	D3	0.91		
	D4	0.90		
Sport habit	E1	0.85	0.93	0.76
	E2	0.89		
	E3	0.91		
	E4	0.88		
	E5	0.84		

The standardized parameter estimates used in the confirmatory factor analysis associated with the sport habit dimension are also listed in Table 3: the factor loading ranges from 0.84 to 0.91; the composite reliability is 0.93; the average variance is 0.76; the composite reliability is >0.60; and the AVE is >0.50, indicating that the internal quality of the model is good, with required composite reliability and convergent validity.

##### Discriminant validity

This study adopts the confidence interval method used by Torkzadeh et al. (2003) to verify if there is any statistical difference in the correlation between two dimensions. As shown in Table 4, none of the correlation coefficients between two dimensions includes 1.0, which means that discriminant validity exists among the dimensions.

**TABLE 4:** Bootstrap correlation coefficients between behavioral intention and sport habit.

		**Bias-corrected**		**Percentile method**	
			
**Parameter**	**Estimated**	**Lower**	**Upper**	**Lower**	**Upper**
Sport habit → Attitude	0.05	0.10	0.27	0.10	0.27
Sport habit → Subjective norm	0.10	–0.20	0.15	–0.22	0.12
Sport habit → Perceived behavioral control	0.10	–0.36	–0.06	–0.35	–0.05
Sport habit → Behavioral intention	0.08	0.58	0.83	0.58	0.83
Attitude → Subjective norm	0.68	0.57	0.79	0.56	0.78
Attitude → Perceived behavioral control	–0.11	–0.26	0.05	–0.27	0.04
Attitude → Behavioral intention	0.51	0.36	0.64	0.36	0.63
Subjective norm → Perceived behavioral control	–0.04	–0.15	0.06	–0.15	0.07
Subjective norm → Behavioral intention	0.67	0.55	0.79	0.55	0.78
Perceived behavioral control → Behavioral intention	0.04	–0.08	0.16	–0.08	0.16

### Hypotheses Testing

The structural equation modeling analysis of this research is conducted with the following indices used by scholars (Fornell and Larcker, 1981; Hooper et al., 2008; Kline, 2011; Hair et al., 2016): the χ^2^ test, the ratio of χ^2^ to the degree of freedom, the goodness-of-fit index (GFI), the adjusted goodness-of-fit index (AGFI), the root mean square error approximation (RMSEA), the comparative fit index (CFI), and the parsimony comparative fit index (PCFI). After modification, the indices of the modeling are shown in Table 5: the GFI is 0.90 (>0.80); the AGFI is 0.87 (>0.80); the RMSEA is 0.05 (<0.08); the CFI is 0.98 (>0.90); and the PCFI is 0.81 (>0.50). All of these values conform to the criteria of model fitness, indicating that the whole results of this research are acceptable.

**TABLE 5:** Overall model fit analysis.

**Fit Indices**	**Allowable range**	**Modified**	**Model fitness**
		**model**	**assessment**
χ^2^(Chi-square)	The smaller the better	217.94	Pass
Ratio of χ^2^ to degree	<3	1.53	Pass
of freedom			
GFI	>0.80	0.90	Pass
AGFI	>0.80	0.87	Pass
RMSEA	<0.08	0.05	Pass
CFI	>0.90	0.98	Pass
PCFI	>0.50	0.81	Pass

**FIGURE 2:**
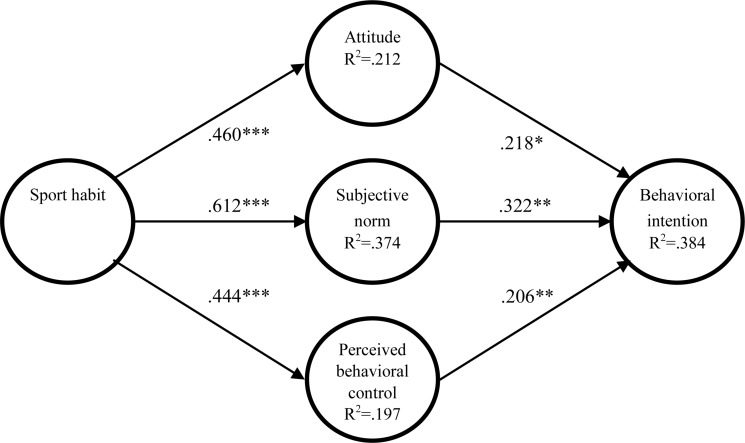
Model diagram of this research.

The result of the analysis is presented in Figure 2 and summarized into Table 6. The result indicates that all the proposed causal relationship is statistically significant at varying degrees of the probability level. The sport habit has significant and positive impact on attitude (β = 0.460, *p* < 0.001), subjective norm (β = 0.612, *p* < 0.001), and perceived behavior control (β = 0.444, *p* < 0.001). Therefore, hypotheses 1, 2, and 3 are supported. Attitude (β = 0.218, *p* < 0.05), subjective norm (β = 0.322, *p* < 0.01), and perceived behavior control (β = 0.206, *p* < 0.01) all have positive impact on behavioral intention; thereby hypotheses 4, 5, and 6 are also supported.

**TABLE 6:** Empirical results of hypotheses.

**Hypothesis**	**Path relation**	**Path value**	**Tenable?**
1	Sport habit → Attitude	0.460***	Yes
2	Sport habit → Subjective norm	0.612***	Yes
3	Sport habit → Perceived behavioral control	0.444***	Yes
4	Attitude → Behavioral intention	0.218*	Yes
5	Subjective norm → Behavioral intention	0.322**	Yes
6	Perceived behavioral control → Behavioral intention	0.206**	Yes

*p < 0.05, **p < 0.01, ***p < 0.001.

From the result, one can see that sport habit is a strong predictor for attitude, subjective norm, and perceived behavior control. Especially in the case of subjective norm, the beta value is.612 and the R^2^ value is.374, both values being the highest of the three paths. On the other hand, the traditional TPB model shows only a moderate level of predictability to behavioral intention. The beta for the three antecedents to behavioral intention are 0.218, 0.322, and 0.206, respectively. The probability level is also slightly less significant ranging from 0.05 to 0.01 levels. Moreover, the three antecedents explain a total of 38.4% of the variance of the behavioral intention (*R*^2^ = 0.384). Although there are no set rules regarding the requisite level of R^2^, past studies using the TPB model do show a high level of predictability to behavioral intention with R^2^ ranging from 0.5 (e.g., Kim and Han, 2010) to 0.7 (e.g., Han and Kim, 2010).

The findings indicate two things. First, when applying the TPB to a different field of study, the level of predictability may vary. This suggests that the TPB is not comprehensive enough to capture every decision-making behavior. In fact, many argue (Han and Kim, 2010; Chang L.-H. et al., 2014) that the TPB needs to be modified in the study of experiential phenomenon. Another finding that is worth mentioning is that subjective norm shows a higher level of susceptibility to sport habit and predictability to behavioral intention than the other two constructs. This potentially suggests that peer opinion is vital for middle-aged and seniors to participate in sport tourism. It also indicates that sport can be a bonding agent in social situation (Baciu and Baciu, 2015).

## Conclusion, Contributions, and Suggestions

### Conclusion

The findings support the hypotheses and the proposed model of this study. Sport habit of the respondents is a valid predictor for their attitude, subjective norm, and perceived behavior control regarding participating in bicycle tourism. The causal relationship is particularly strong between sport habit and subjective norm. The three antecedents also show moderate impact on behavioral intention.

The R^2^ for behavioral intention is relatively low compared to past studies (e.g., Han and Kim, 2010; Kim and Han, 2010). One of the reasons is that previous studies often add other predictors to aid the predictability of the TPB model. This study supports these previous studies (i.e., lack of additional predictors result in a low R^2^ value).

There is one more thing to note. The sample size of this study is relatively small for structural equation modeling, but the researchers did assert that the size of 150 responses is adequate for a model that is composed of less than seven constructs (Hair et al., 2016). Our sample size, 210, is bigger than 150, so it is still adequate for the tests of this study. Nevertheless, small sample size is one of the limitations of this study. This is due to the difficulty of identifying eligible respondents and enlisting their participation in the study. Thankfully, the proposed model only consists of five constructs. However, it is still preferable to have a larger sample size. For instance, the sample actually comprises of very few seniors (individual over 75). Furthermore, more than half of the respondents are not frequent participants of the sport. These limitations can affect the result of this study, which should be taken into account.

### Theoretical Contributions

As mentioned in the previous section, this study makes contribution to tourism literature and theory because it extends the TPB model by adding a new factor of sport habit and taking the middle-aged and seniors as the research subjects to investigate their behavioral intention of participation in bicycle tourism.

These findings allow the study to posit a theory. Respondents’ sport habit works best when they have companions. Most, if not all, sports are social event (Baciu and Baciu, 2015). Especially for an adult whom competing is a less important incentive, socialization can replace the desire to win as the main motivation to engage in sport. Among other factors, sports can be responsible for the health of adolescents, and it improves not only their physical health but also their socialization, as emphasized by Coakley (2011) and Giulianotti (2004) who believe that sports have a great potential to help solve problems and improve the quality of life of individuals and communities. This is also true in the case of middle-aged and seniors.

### Empirical Suggestions

This research aims at discussing the behavioral intention of the participation of the middle-aged and seniors in bicycle tourism, and further investigates the influential factors of behavioral intention. At the end of the paper, constructive advice is provided for the middle-aged and senior participants as well as the relevant authorities of sport tourism, based on the empirical analysis results. Furthermore, several suggestions are presented, in the hope of shedding light on the future studies of sport tourism. Based on the research results, the following suggestions are proposed for reference.

First, for the middle-aged and senior participants of bicycle tourism, hypothesis 1 of this research implies that if the middle-aged and seniors perceive bicycle tourism positively, they are more likely to take part in this activity. Therefore, it is advised that they should cultivate a positive and optimistic life attitude. As an old Chinese saying goes, “Eating appropriately is better than taking any medicine; exercising regularly is better than having herbal tonics of any kind.” After all, people need to exercise to stay active and alive. The proper cognition of “being active” is an important factor for the middle-aged and seniors in their stages of life. Hence, it is proposed that they should consult with others or take their own life patterns into account to develop interests in suitable sports, and acquire further understanding of them. Sport for older generation should be sustainable and safe as slow sport is. For example, Chiu (2011) lists four principles for selecting appropriate sports for seniors: (1) Many senior participants in sports have different physical restrictions; hence, trainers need to assess their physical conditions to design their exercise prescriptions, such as weight-bearing or non-weight-bearing, and low-intensity or medium-intensity exercises. (2) Sports like cycling, swimming, chair, or floor exercises are appropriate options. Dancing and water aerobics are also favorable selections. However, trainers should still pay heed to the safety, fun, and feasibility of those sports. Most important of all, seniors should engage in these sports and be motivated to continue. (3) Individualization, personal uniqueness of the seniors, diversity, and trainees’ interests should all be considered in selecting the exercise style. (4) Exercise prescriptions should include the improved style of muscle-stretching and strengthening activities as well. From the above discussion, there are a variety of exercises that are suitable for the middle-aged and seniors, such as bicycle tourism, which was studied in this research. Besides its lower intensity, its combination of sports and tourism allows seniors to enjoy both at the same time, thereby elevating their satisfaction in such aspects as physiology, psychology, social networking, and relaxation. Therefore, it is advised that the middle-aged and seniors should try their hand at bicycle tourism after obtaining some understanding about it. Through the participation in bicycle tourism, participants can come to perceive this tourism pattern more positively, and their willingness to continue performing it will be enhanced. The research results also show that the subjective norm of the middle-aged and seniors for participating in bicycle tourism has a significant influence on their behavioral intention. It suggests that they need the encouragement and support of their friends and family to do the exercises. However, the possible dangers of seniors’ engaging in outdoor sports, most of the time, have been highlighted due to some sporadic accidents reported by the media, triggering concerns and impeding their involvement in outdoor sports. Therefore, it is recommended that the middle-aged and seniors should select exercises appropriate for their physical condition. Through safe and smooth engagement in exercises, friends and family will accordingly recognize the benefit of the outdoor sports, so the senior participants can have more opportunities to take part in more diversified sport tourism.

Second, for organizers of the bicycle tourism of middle-aged and seniors, based on the research results, the influence of perceived behavioral control on behavioral intension is significant. Therefore, in order to encourage the participants to become more involved in the tour, it is recommended that the organizers of bicycle tourism provide the middle-aged and seniors with comprehensive information about the riding and routes, and that they should pay close attention to the situations of flat tire or other mechanical malfunction incidents that might occur en route. The difficulty level of activities should also be taken into account while designing bicycle tourism for the middle-aged and senior riders, since especially head winds and slanting slope of roads can be burdensome to them. Besides investigating the participants’ age distribution and physical conditions in advance, it is recommended to appoint leading cyclists at the front and escorts at the back of the bike team. Furthermore, field surveys of event venues and rest stops should be conducted beforehand in order to avoid inappropriate sections of the road, thereby strengthening the safety of participation. Certainly, it is crucial that the organizers possess risk management ability. They have to be aware of the physical conditions of the middle-aged and seniors, and handle emergencies by making prompt and appropriate handling, such as CPR or heatstroke treatment. With the deliberate and comprehensive arrangement, the middle-aged and seniors will feel more secure and comfortable. After experiencing the positive results of bicycle tourism, they will have higher motivation for rejoining similar activities.

### Suggestions for Future Research

In reviewing the literature, it is found that previous relevant research on sport tourism, which is conducted according to the TPB, mainly focuses on marine activities. Since the leisure characteristics of marine activities differ from those of terrestrial activities, the theoretical contribution of this research is to expand the application of the TPB, providing future research on the TPB with valuable reference data for comparison. Because of the aging population in Taiwan, the sport issues of the seniors have been highly valued in recent years. Therefore, the analysis of bicycle tourism in this research can not only offer the middle-aged and seniors with diversified activity options but also provide a practical and valuable reference for relevant research on sport tourism and exercises for senior adults in the future.

As for future research, it is worth noting that the influence of sport habits on behavioral intention did not reach the significant level after it is integrated into the model for investigation. This result might imply that more influential factors are involved in the participation of the middle-aged and seniors in bicycle tourism. Therefore, further investigations are recommended, especially on the physical aspects involved in bicycle tourism, as these are more complicated than other simple activities, such as taking a stroll. Finally, future research could be undertaken to investigate if muscle endurance, cardiovascular function, or even physical coordination and sense of balance plays an influential role in the participation of the middle-aged and seniors in bicycle tourism.

## Data Availability Statement

All datasets generated for this study are included in the article/supplementary material.

## Ethics Statement

Ethical review and approval was not required for the study on human participants in accordance with the local legislation and institutional requirements. Written informed consent from the patients/participants legal guardian/next of kin was not required to participate in this study in accordance with the national legislation and the institutional requirements.

## Author Contributions

S-WL made substantial contributions in developing the research framework, designing the methodology, conducting the survey, writing the manuscript, and searching for references. S-YH and M-YL made substantial contributions in conducting the survey, writing the manuscript, and searching for references. J-LH made substantial contributions in designing the methodology, revising the manuscript, and writing the authors’ response notes.

## Conflict of Interest

The authors declare that the research was conducted in the absence of any commercial or financial relationships that could be construed as a potential conflict of interest.
